# Japanese translation and cross-cultural validation of the Adult Social Care Outcomes Toolkit (ASCOT) in Japanese social service users

**DOI:** 10.1186/s12955-019-1128-7

**Published:** 2019-04-11

**Authors:** Hiromi Nakamura-Thomas, Mie Morikawa, Yoko Moriyama, Takeru Shiroiwa, Makoto Kyougoku, Kamilla Razik, Juliette Malley

**Affiliations:** 1 Graduate School of Health, Medicine and Welfare, School of Occupational Therapy, Saitama Prefectural University, 820 San-no-miya, Koshigaya city, Saitama Prefecture 343-8540 Japan; 2 Department of Policy Studies, Tsuda University, 1-18-24 Sendagaya, Shibuya ward, Tokyo, 151-0051 Japan; 3 National Institute of Public Health, 2-3-6 Minami, Wako city, Saitama Prefecture 351-0197 Japan; 4 Graduate School of Health Science and Social Welfare, School of Occupational Therapy, KIBI International University, 8 Iga town, Takahashi city, Okayama Prefecture 716-0018 Japan; 5 Personal Social Services Research Unit (PSSRU), University of Kent, Cornwallis Building, George Allen wing, Canterbury, Kent CTs 7NF UK; 6 Personal Social Services Research Unit (PSSRU), London School of Economics and Political Science, Houghton St, London, WC2A 2AE UK

## Abstract

**Background:**

The aim of this study was to develop and perform cross-cultural validation of a Japanese version of the Adult Social Care Outcomes Toolkit (ASCOT) four-level Self-Completion questionnaire (SCT4) instrument to measure Social-Care Related Quality of Life. It was important to develop a Japanese version of the ASCOT-SCT4 and validate it in the Japanese context, given the interest in measuring outcomes of social care services in Japan.

**Methods:**

The original version of ASCOT-SCT4 was translated into Japanese following good practice guidelines. Additionally, comments and feedback were obtained from an independent committee engaged in managing and providing social care services to refine the flow of sentences of the newly developed translated version. The resulting version was tested for cross-cultural validation among community-dwelling adults who use social care services to confirm the factorial structure and the scale system of the Japanese version, using Structural Equation Modeling and Item Response Theory.

**Results:**

Vigorous discussion was needed to translate the original version into Japanese especially for the items *control over daily life* and *dignity*. These two items were linguistically difficult to express in everyday language so potential participants could easily understand the intended concepts. In the cross-cultural validation, we obtained values for model fit within the acceptable range: between 0.706 and 0.550 for factor loadings, 0.923 for the Comparative Fit Index, 0.910 for the Tucker-Lewis Index, and 0.083 for the Root Mean Square Error of Approximation. This confirmed the factorial structure of the Japanese version. The IRT analysis, however, revealed that the scale system needed refinement to facilitate appropriate differentiation between each response option.

**Conclusions:**

This study provided preliminary evidence that the Japanese version of ASCOT-SCT4 is valid. As a result, the Japanese version was finalized and approved by the instrument developer.

## Background

Provision of social care services was introduced in Japan in 2000. Its aim is to maintain living in the community with dignity as long as possible [1]. Social care services are generally expected to help to sustain and/or improve the Quality of Life (QoL) among service users. Developing outcome measures that reflect service users’ perspectives is essential to assess social care services appropriately [2]. However, there was no standardized method for capturing the QoL of service users and attributing differences or changes in QoL to the actions of social care services. Measurements of health-related QoL and specific diseases developed in the fields of health, medicine and epidemiology have been used in social care in Japan although these are less suitable because they conceptualize QoL too narrowly [3–5]. Capability-based assessments and satisfaction questionnaires are used internationally [6]. More recently, the Adult Social Care Outcomes Toolkit (ASCOT), a Social-Care Related QoL (SCRQoL) outcome measure, was developed [7]. Attention has been drawn in Japanese reports to the potential of the ASCOT to assess Japanese social care services as the questions capture both aspects of decision-making and satisfaction with social care services [8–11]. However, prior to the current study an approved Japanese version of the ASCOT instrument, has not been developed.

In Japan, people aged 65 and over are referred to as older adults as 65 is the official retirement point. Social care services in Japan are provided for both older and younger adults. The vast majority of the service users, however, are people aged 75 and over. The group of people is increasing rapidly and steadily [1]. The ACOT-SCT4 is most appropriate for this group, hence the focus on this study on translating and validating the ASCOT-SCT4 as opposed to other ASCOT measurements.

The ASCOT-SCT4 is a multi-attribute utility (MAU) instrument, consisting of a multi-attribute descriptive system and a set of values based on a stated preference study using a mixture of Best Worst Scaling and Time Trade Off [7]. The ASCOT-SCT4 consists of eight attributes of quality of life which are captured in nine questions: *control over daily life*, *personal cleanliness and comfort*, *food and drink*, *personal safety*, *social participation and involvement*, *occupation*, *accommodation cleanliness and comfort*, and *dignity* [7]. There are four- response levels in each item representing four outcome states, from the best to the worst: ‘ideal state,’ ‘no needs,’ ‘some needs’ and ‘high-level needs’ [12]. A Dutch version of the ASCOT-SCT4 has been developed [13]. The English and Dutch versions of the ASCOT-SCT4 have been validated [14, 15] and has gained international attention as a promising instrument for the evaluation of social care services [6]. The ASCOT-SCT4 is recommended for analysis of the cost-effectiveness of social care services [11]. Cost-effectiveness of social care services is an important viewpoint for municipalities in Japan [2] as they are in charge of planning, providing and evaluating the services [1].

To obtain social care services in Japan, registration at a municipality office is required which includes an assessment on the Activities of Daily Living (ADL) and Instrumental Activities of Daily Living (IADL) measures. The performance levels are categorized in to one of eight levels of care need: no need (independent), support 1, support 2, care 1, care 2, care 3, care 4, and care 5. The content, frequency and intensity of services are capped according to the levels of care needs. For instance, people registered for support level 1 are considered able to perform ADLs independently but need support for IADLs, thus, they receive support with cleaning, cooking, grocery shopping, and managing financial matters and medication. People registered for support level 2 receive home modification services (renting equipment and/or subsidies) and more intensive and more frequent services than people registered for support level 1. People registered for care levels are considered to have difficulties performing ADLs without help, for instance, people registered for care level 1 have basic mobility difficulties, such as standing and walking. People registered for care level 2 have mild difficulties performing ADLs, and people registered for care level 3 are unable to perform ADLs without almost full assistance. Thus, they receive nurse home visits, home-based rehabilitation services, bathing services at home, day care services, short stay services and home modification services, including installing handrails in bathrooms and corridors. The intensity, duration and frequency of service delivery is higher for care level 3 than care level 1 or 2. People registered for care level 4 require full assistance performing ADLs. Short-stay services are recommended for the people to give their caregivers a break from caring. People registered for care level 5 are totally dependent with regards to performing any ADLs and those people tend to have difficulty communicating [1].

The provision of social care services in Japan is designed to promote home-based care, not institutional care. The provision aims to reduce the caregiving burden; however, socialized care in Japan is still heavily reliant on familialism, defined as an emphasis upon the family as the primary locus of welfare provision by way of intra- and inter-generational mutual aid. Service users are institutionalized when their family caregivers are incapable of taking care of them [16]. A study compared 18 countries to identify characteristics of countries with familialism, including Japan, regarding welfare state characteristics [17]. In the study, some differences between Japan and the UK were shown as follows: Japan took the first place for age-bias of social spending programmers while the UK took the 11th place. Japan took the 13th place for the percentage of government spending on social services towards Gross Domestic Product (GDP), meaning Japanese government less spends on the services, while the UK took the 5th place. Japan took the second place for the percentage of elderly people living with their children while the UK took the 7th place [17].

Given the interest in measuring outcomes of social care services in Japan, it is important to develop a Japanese version of the ASCOT-SCT4 and validate it in the Japanese context. Thus, the aim of this study was to develop and perform cross-cultural validation of a Japanese version of the ASCOT-SCT4 instrument.

## Methods

### Design and setting

In this study, we translated the ASCOT-SCT4 for use in Japan following good practice guidelines for translation of patient reported outcome measures. The translation was carried out in collaboration with the measure developer, members of the Personal Social Services Research Unit (PSSRU) at the University of Kent, United Kingdom. The University of Kent retains copyright in the Japanese translated version. To carry out the translation process, an agency in Chicago, USA, with a plenty of experience translating measurement instruments in the health care field was employed. Using the translated version, we conducted a survey of community-dwelling people in receipt of social care services to verify the design of the original ASCOT-SCT4 in the Japanese context for this particular population.

The survey targeted Japanese community-dwelling people in receipt of social care services. Based on the characteristics of service users in Japan, the sample was expected to be dominant by people aged 75 and older. People in residential care homes people were excluded from the survey. Written informed consent was obtained from all study participants. The research protocol was reviewed and approved by the Research Ethics Committee in Saitama Prefectural University (SPU-IBRA #26105) (first author’s employer) and the National Institute of Public Health (NIPH-IBRA#12123) (the third author’s employer).

### Overview of the translation processes

The translation process was conducted in close cooperation with the measure developer -- members of the Personal Social Services Research Unit (PSSRU), University of Kent, UK and the agency. The ASCOT-SCT4 was translated into Japanese following two international guidelines: Consensus-based Standards for the selection of health Measurement Instruments (COSMIN) taxonomy [18] and The ISPOR Patient-Reported Outcomes Translation and Linguistic Validation Good Research Practices Task Force Report [19].

Table 1 shows the translation process, employed in this study, based on the international guidelines. There were nine steps. In each step, reports were written which were submitted for review by the developer in Step 10. Step 1 was the initial translation. Two bilingual translators who were Japanese native speakers fluent in English independently translated the ASCOT-SCT4 questionnaire into Japanese. The translators were guided by a concept elaboration guide which contains definitions of key item concepts. Step 2 was the first synthesis. The two translations were then harmonized into one version by the two translators. The Japanese research team members reviewed the translation and discussed any concerns over word choice with the translators. This was an iterative process which aimed to ensure that the translation used everyday language familiar to social care service users. A synthesized version was produced which was back-translated into English. Step 3 was the first back translation. Two other bilingual translators, blind to the original version independently produced back translations into English. The two back translations were reviewed by the Japanese research team to check that they reflected the meaning and concepts of the original English version. Members of the developer team provided feedback and revisions to the synthesized forward translation and then the back translations were made.

**Table 1:** Translation process, employed in the current study

Step 1: Initial Translation	- two translations (FT1 &FT2) - into target language - informed + uniformed translator	Written report for each version (FT1 & FT2)	Step 10: submission and appraisal of all written reports by developer
Step 2: 1st Synthesis	- synthesize FT1 & FT2 into FT-12 - resolve discrepancies with translators’ reports	Written report	
Step 3: 1st Back Translation	- two English first-language - naïve to outcome measurement - work from FT-12 version - create 2 back translations FBT1 & FBT2	Written report for each version (FBT1 & FBT2)	
Step 4: 2nd Translation	- two NEW translations (ST1 & ST2) - into target language - informed + uniformed translator	Written report for each version (ST1 & ST2)	
Step 5: 2nd Synthesis	- synthesize ST1 & ST2 into ST-12 - resolve discrepancies with translators’ reports	Written report	
Step 6: 2nd Back Translation	- two NEW English first-language - naïve to outcome measurement - work from ST-12 version - create 2 back translations SBT1 & SBT2	Written report for each version (SBT1 & SBT2)	
Step 7: Expert committee review	- methodologist, developer, language professional, translators - review all reports - reach consensus on discrepancies - produce pre-final version	Written report	
Step 8: Pretesting	- cognitive debriefing - probe to get at understanding of item/paraphrasing	Written report	
Step 9: Independent committee review	-inviting public officers and social care practitioners -review the results of the cognitive debriefing to refine the pre-final version	Written report	

*FT* First Translation, *FBT* First Back Translation, *ST* Second Translation, *SBT* Second Back Translation

Step 4 was the second translation. Two newly employed bilingual translators who were Japanese native speakers fluent in English independently translated. The methods employed in Step 4, 5 and 6 were the same performed in Step 1, 2 and 3 respectively. In Step 6, two newly employed bilingual translators, blind to the original version independently produced back translations into English.

Step 7 was the expert committee review, inviting specialists listed in Table 1, to review all translation reports, created in the previous steps. Any concerns over meaning were discussed until a consensus was reached. The translation was revised to be tested in the cognitive debriefing (Step 8) based on the feedback from the committee.

Step 8 was the cognitive debriefing. Cognitive debriefing is a qualitative research tool used to determine whether respondents understand items and concepts behind them in an instrument in the way it is intended [20]. Cognitive debriefing interviews involve using follow-up questions to gain a better understanding of how respondents interpret questions. In this project participants were chosen based on theoretical sampling [21], with the following factors guiding the selection: gender, age, length of time in education, living area (city centre or an urban area in the city of Tokyo, Japan) and the level of social care need. According to the recommendation from the developer members, five participants were selected.

The characteristics of each participant were as follows: the first participant (P1) was a 96-year-old woman with 8-years education, living in a suburban area. She was registered Care level 2, obtaining a bathing service at a nursing facility. The second participant (P2) was a 77-year-old man with 9-years education, living in a suburban area. He was registered Care level 1, obtaining a bathing service at a nursing facility. The third participants (P3) was a 67-year-old man with 16-years education, living alone in a city centre area. He was registered Support level 1, monitored by public health nurses. The fourth participant (P4) was an 83-year-old man with 12-years education, living in a city centre area. He was registered Support level 2, obtaining housekeeping services, mainly meal services. The fifth participant (P5) was a 73-year-old woman with 12-years education, living in a city centre area. She was applying for registration with social care services but had not yet been registered when the interview was conducted.

The participants were asked whether they understood each sentence in the translation, and whether they were able to paraphrase them. Participants were individually invited to their newest community centres to conduct the cognitive debriefing. Prior to the interviews, a training session was provided by the translation company via the telephone for the first and second author who were in charge of data collection. Participants’ responses were written down during the interviews, and then translated into English by the Japanese team members. They were interviewed with each interview taking approximately 30–40 min.

Step 9 was an independent committee review. This step is not included in the international guidelines, but the Japanese research team thought it was important to add to gather feedback from stakeholders to help refine the pre-final version. The committee were comprised of seven members including public officer and managers of social care providers in the municipality, located south end of the metropolitan area. Six of them worked in community-based social care service providers. One worked at a municipality office as a service manager overseeing the registration of new service users. They were asked to provide their feedback and opinion on, how natural and everyday language used in the pre-final version was, and how easy they thought it would be for community-dwelling service users to understand and respond to the pre-final version. The refined version was submitted to members of the ASCOT development team, with explanations of the changes made. The developer members, Japanese research team and the agency were all engaged in the final committee review at Step 10 to confirm the pre-final version.

### Examination of the pre-final version

Questionnaires were distributed to social care service users in a municipality, located north-west from Tokyo. The municipality and the Japanese research team had an agreement to collaborate to improve social care services. The ageing index, the percentage of people who are 65 and older as compared with the total population, was 26.8% in the municipality, which is very close to the average national Fig. (26.7% in 2016). The municipality administers surveys at regular intervals. The department of social care services in the municipality estimated that 2370 would be possible distribution number. The department included the translated ASCOT-SCT4 to their regular survey. To ensure data protection, the department and the Japanese research team developed an agreement to share the collected data. The survey included a range of questions, covering sex, age, living situation (living alone or not), care need level (no need, support 1, support 2, care 1, care 2, care 3, care 4, and care 5), and self-rated health (very good, good, fair, or poor) in addition to the translated ASCOT-SCT4.

To understand the overall characteristics of responses to the translated ASCOT-SCT4, we analysed the descriptive data for the items and response levels of the measure (‘ideal state,’ ‘no needs,’ ‘some needs’ and ‘high-level needs’). Japanese care need levels are often combined into three groups (support level 1 and 2; care level 1 and 2; care level 3, 4 and 5) in nationwide survey reports [1]; thus, this grouping was employed in this study. Associations between ASCOT-SCT4 items and the three groups were assessed using Cramer’s V, which indicates how strongly two categorical variables are associated, with valued of one indicating a strong association or no association [22]. A value of 0.10, 0.30, and 0.50 is considered to be a small, medium, and large effect size, respectively [23]. Analysis was carried out in Statistical Package for the Social Sciences version 21 in Japanese (SPSS, Inc., Chicago, IL, USA).

The factorial structure and the scale system were investigated using factor analysis and an Item Response Theory (IRT) approach respectively to verify the design of the original ASCOT-SCT4 in the Japanese context. These methods are beneficial for investigating the dimensionality and deciding on the definitive selection of items per dimension [18]. These methods are also useful for investigating the validity and reliability of an instrument [24]. For the Dutch version the researchers explored the test-retest reliability and found that the following four items showed a substantial level of reliability: *control over daily life*, *personal cleanliness and comfort*, *social participation and involvement*, and *accommodation cleanliness* [13]. Since we had only cross-sectional data, our approach was to use IRT to examine whether participants were able to differentiate between the response levels. Following the developers of the original measure [7], we also examined the inter-item relationships with the use of polychoric correlations and calculated Cronbach’s alpha coefficient to assess the internal consistency of the scale. A value between 0.70 and 0.95 is considered ‘good’ [25].

To investigate the factorial structure of the Japanese version both Exploratory Factor Analysis (EFA) and Confirmatory Factor Analysis (CFA) were performed. In the EFA, we assessed whether the one factor structure of the original measure [7] was identified in the Japanese version. We used a contribution ratio of the factor of above 20% [26]. Both the CFA and EFA were needed to confirm the robustness of the factorial structure in the same study [27]. With the CFA, we used Structural Equation Modeling (SEM), a comprehensive statistical analysis combining path analysis and factor analysis [27]. We used a robust weighted least squares estimation with missing data and with no missing data. Model fit indices included factor loadings, path coefficients, Comparative Fit Index (CFI), Tucker-Lewis Index (TLI), and Root Mean Square Error of Approximation (RMSEA). No limit was set to obtain those values in the two factor analysis in this study. A value above 0.95 for the CFI and TLI was considered a good fit and a value below 0.08 for the RMSEA indicated a good fit [28]. Factor loadings and path coefficients were also analysed in this study to examine the model fit.

To investigate the scale system, the response levels in the Japanese ASCOT-SCT4, we used IRT, with maximum likelihood estimation and robust standard errors. An advantage of the IRT approach is the fact that item and scale properties are not sample dependent. Thus, item and scale properties are the same even when the analysis is executed using data from different populations or measurement conditions [22, 24]. IRT approaches also lend themselves to visual presentation, such as graphs [18]. To explore validity, we investigated item discrimination and item difficulty. The item discrimination parameter indicates how well items identify respondents at different levels of the latent variable. The value range is from 0.5 to 2.5. Item difficulty indicates how difficult it was to achieve a 0.5 probability of a correct response for a specific item given the respondent’s score on the latent variable. The values in this analysis range from ―4.0 to 4.0 [29]. To explore reliability, we focused on the Item Response Category Characteristics Curve (IRCCC), as it is determined by relative relationships among the scales, showing how well the scales work for each item. A steep IRCCC means that the item discrimination is high and differences in response probabilities between the ordinal categorical data are larger, suggesting higher reliability. A flatter IRCCC means that the item discrimination is low and differences in response probabilities between the ordinal categorical data are smaller, suggesting lower reliability. A curve shifted to the left means the item is relatively easy while a curve shifted to the right means the item is relatively difficult [26].

For the EFA we used the Statistical Package for the Social Sciences version 21 in Japanese (SPSS, Inc., Chicago, IL, USA). *M*plus version 7.4 (Muthén and Muthén, Los Angeles, CA) was used for the CFA and IRT analysis. For establishing a Path diagram, we used the Ωnyx version 1.0 (University of Virginia & Max Planck Institute for Human Development) [30]. For the graphical data representation of the IRT analysis, we used the Exametrica version 5.3 [31].

### Creating the final version

Upon review of this study’s results, a final Japanese version of the ASCOT SCT4 was created, and approved by the developer of the original measure.

## Results

### Translation processes

Some changes were made, mainly to improve the flow of the sentences in Japanese. There were three question items which required detailed discussions around word choice and meaning: *control over daily life* and two *dignity* questions. These were linguistically difficult for non-English speaking people, including participants in the cognitive debriefing, the independent committee and the researchers. They were also difficult to translate and required much discussion between the agency, the Japanese research team and the developer of the original instrument.

Considering the word ‘control,’ in colloquial Japanese, the word ‘control’ is used as a technical term, such as ‘the machine is under control’ and ‘he controls the vehicle’. Following discussion, however, the direct translation of ‘control’ was employed with the description of the words written in the original version. The description is as follows: *control over daily life* means ‘having the choice to do things or have things done for you as you like and when you want’. Since the word and its meaning have been seen in the media, we expected it to be understood by participants.

The word ‘dignity’ is often used in palliative care and for people with cognitive impairment in Japan. The concept of *dignity* is defined in the ASCOT as ‘the negative and positive psychological impact of support and care on the service users’ personal sense of significance’. The *dignity* questions aim to capture the effects of care and how the person is treated on subjective well-being, i.e. utility derived from the process of care. The word ‘self-esteem’ seemed to be more appropriate to reflect this meaning in Japanese. We employed the word ‘self-esteem’ in the dignity questions during cognitive debriefing.

There were discussions regarding some of the other items. For instance, for Japanese people the notion of personal safety includes being involved in natural disasters, such as earthquakes and floods. However, the concept aims to capture the respondents’ perceptions about safety both inside and outside the house, thus, no additional description was needed.

Table 2 shows the responses in the cognitive debriefing (Step 8). Issues identified in the *control over daily life* and two *dignity* question items. In terms of *control over daily life*, four participants understood and paraphrased it, while, one participant could neither understand nor paraphrase it. The four participants, however, reported “something is wrong with ‘control’ for daily life” as the expression is not commonly used in everyday language. Moreover, daily life is not perceived as an area that could be controlled in Japanese culture. One participant, therefore, complained that “it sounds like there is a commander who controls me.” By providing the following description “By ‘*control over daily life*’ we mean having the choice to do things or have things done for you as you like and when you want,” all participants understood the question.

**Table 2:** Responses in the cognitive debriefing process. Used with permission from the University of Kent. All rights reserved

Question item	Question sentences for the cognitive debriefing	Understand	Words or phrases difficult or upsetting	Feedback comments
Control over daily life	**Back translation** How much control you have over your daily life? **Sentence to be presented** あなたは日常生活をどのくらいコントロールできていますか?	P1: No P2: Yes P3: Yes P4: Yes P5: Yes	P1, P3, P4, P5: control	P3: I understand the word and the meaning of “control” but it would be better if you use “統制” or “統御”. The word seemed to be the direct pronunciation. P4: If the word “control” is changed into “統制” or “統御”, it is still not our everyday conversation word. P5: we do not use the word “control” in terms of our daily life. The word sounds as if it was a command.
Dignity	Dignity filter question **Back translation** How has getting help to do things affected your self-esteem? **Sentence to be presented** 何かをする際に助けを得ることはあなたの自尊心にどのような影響を及ぼしますか?	P1: No P2: Yes P3: Yes P4: Yes P5: Yes	P1, P2, P4, P5: self-esteem	P2, 4 & 5: I understand the word and the meaning of “自尊心” but I can’t paraphrase it.
	ASCOT Dignity question **Back translation** How the way you are helped and treated affected your self-esteem? **Sentence to be presented** 支援のされ方や扱われ方はあなたの自尊心にどのような影響を及ぼしますか?	P1: No P2: Yes P3: Yes P4: Yes P5: Yes	P1, P2, P4, P5: self-esteem P3 & 4: treated P5: The whole sentence	P3: “扱われ方” sounds like I am an object. I prefer “対応の方法”. P5: The sentence sounds unnatural.

In terms of the *dignity* questions, one participant could not understand and paraphrase them. Two participants understood the meaning of the word ‘self-esteem,’ however, they could not paraphrase it. Another participant commented that the word ‘treated’ made him feel as if he was treated as an object. The word was changed to ‘treated with respect.’

At an independent committee (Step 9), the words ‘control over’ for the *control over daily life* item and ‘self-esteem’ for the *dignity* questions were also discussed. The description in the original version was that “‘by control over daily life,’ we mean having the choice to do things or have things done for you as you like and when you want.” The question was revised as follows to reflect the concept behind the original question: “How much are you able to decide by yourself in terms of your daily life? Please think about the cases, including another person performing according to your decision.” The response options were revised accordingly. For instance, for the ‘ideal state’ in the four level system, the following sentence was employed: ‘I decide based on my preference.’

In the two *dignity* questions, a number of comments were provided regarding the word ‘self-esteem,’ as the word could be misunderstood. For instance, the word had the meaning of ‘being proud of something one is good at’ in Japanese. Moreover, the meaning inferred a certain negative impression in older adults because in Japanese culture people tend to hesitate to show one’s strength in public. The following sentences were used in each question item: ‘How do you feel about yourself in terms of getting care and support?’ for the *dignity* filter question and ‘How you do feel in terms of the treatment when getting care and support?’ for the ASCOT *dignity* question.

Across the nine question items, the independent committee criticized the length of response sentences. For instance, parts of the question are repeated in the response options. In a previous measure of quality of life in Japan, the response options were shorter, e.g. ‘completely satisfied’, ‘moderately satisfied,’ ‘moderately dissatisfied, and ‘completely dissatisfied.’ Thus, the committee recommended shortening the response options, but this was not done as it was felt it would stray too far from the English version.

All submitted reports were reviewed and discussed with the members of the ASCOT developer team and the Japanese research team (Step 10). In conclusion, the original form of the original measure was retained. A pre-final version of the translation was agreed and used within a survey for subsequent cross-cultural validation.

### Examination of the pre-final version

A total of 1141 questionnaires were completed, yielding a response rate of 48% among the total distribution number, seemingly obtaining the enough number for data analysis. The following cases were excluded from the analysis: cases missing answers to all the ASCOT questions and missing care level information. Thirty-nine cases were excluded, which brought the total number of cases for the analysis to 1102. Table 3 shows the demographics of the participants in the examination of the pre-final version. Among the participants, 64% were women. Over 80% of the respondents were 75 years of age or older, whereas 16.6% were 65 to 74 years old. There were 189 participants (17.2%) who lived alone. Recipients of Care level 1 constituted 21.9% of the sample, followed by recipients of Care 2 level (20.2%), Support level 2 (18.9%). Over half of the respondents rated their health as good (53.0%); under a third rated their health as fair (29.0%) and 6.2% as poor. Given the distribution of care need levels within the sample, the care need levels of participants in the cognitive debriefing seemed to cover the diversity of survey participants. A notable difference in that the survey data were collected only from people living in a suburban area while the data for the cognitive debriefing were collected from people living in both suburban and city centre areas.

**Table 3:** Participants’ demographics for examining the pre-final version

	*n* (%)
Total	1102 (100)
Gender	
Men	374 (33.9)
Women	705 (64.0)
Missing	23 (2.1)
Age group (years old)	
45–64	14 (1.3)
65–74	182 (16.5)
75 and older	891 (80.9)
Missing	15 (1.4)
Living alone	
Yes	189 (17.2)
No	865 (78.5)
Missing	48 (4.3)
Care need level^a^	
Support level 1 & 2	342 (31.1)
Support level 1	134 (12.2)
Support level 2	208 (18.9)
Care level 1 & 2	464 (42.1)
Care level 1	241 (21.9)
Care level 2	233 (20.2)
Care level 3–5	261 (23.7)
Care level 3	124 (11.3)
Care level 4	83 (7.5)
Care level 5	54 (4.9)
Uncertain	3 (0.3)
Missing	32 (2.9)
Self-rated health status	
Very good	35 (3.2)
Good	584 (53.0)
Fair	320 (29.0)
Poor	68 (6.2)
Missing	95 (8.6)

^a^Care need level under Japanese social care system

Table 4 shows the responses to the ASCOT-SCT4 item and scale level. The percentage of valid responses ranged from 93.4 to 98.7%. For each ASCOT-SCT4 item, the median score was around 2.0, and the modal response option was the ‘no needs’ level (39.3 to 63.3%). Each item yielded responses to each of the four levels, but the ‘high-level needs’ received the lowest number of responses (0.8 to 12.1%).

**Table 4:** Response to the ASCOT items and scale level

Question item	Valid *n*	%^a^	Mean	SD	MED	DIS	SKE	KUR	Scale level %^b^			
									Ideal state	No needs	Some needs	High-level needs
Control over daily life	1053	95.6	2.2	0.8	2.0	0.67	0.23	−0.54	21.56	45.39	28.11	4.94
Personal cleanliness	1087	98.6	1.8	0.6	2.0	0.35	0.36	0.74	30.91	62.19	6.07	0.83
Food & drink	1080	98.0	1.8	0.7	2.0	0.43	0.56	0.77	29.35	59.35	9.53	1.76
Personal safety	1088	98.7	1.9	0.8	2.0	0.60	0.52	−0.19	32.08	47.98	17.19	2.76
Social participation	1074	97.5	2.4	0.8	2.0	0.63	0.27	−0.35	10.34	48.60	31.75	9.31
Occupation	1054	95.6	2.4	0.9	2.0	0.80	0.10	−0.73	15.37	39.28	33.21	12.14
Accommodation cleanliness	1078	97.8	1.9	0.6	2.0	0.41	0.43	0.74	23.01	63.27	12.15	1.58
Dignity filter question	1030	93.5	2.0	0.7	2.0	0.46	0.28	0.04	21.07	58.83	18.64	1.46
ASCOT dignity question	1029	93.4	1.9	0.7	2.0	0.47	0.31	−0.13	25.85	56.27	16.72	1.17

*SD* Standard Deviation, *MED* Median, *DIS* dispersion, *SKE* Skewness, *KUR* Kurtosis, ^a^frequency (%) in the total number of the participants,^b^ frequency (%) in the valid number of the responses in each domain

Table 5 shows the percentage of valid response for in each scale level of the Japanese version by care level groups. A number of significant associations, measured by Cramer’s V, were observed between care levels and ASCOT for the following items: *food and drink* (0.087, *p* < 0.01)*, social participation and involvement* (0.108, *p* < 0.001)*, occupation* (0.158, *p* < 0.001)*, accommodation cleanliness and comfort* (0.083, *p* < 0.01), and two *dignity* items (0.079 and 0.081, both *p* < 0.05). Values for the effect size were small.

**Table 5:** Percentage of valid responses for each scale level in the Japanese ASCOT-SCT4 version by care level groups

Question item	Scale level	Care level group^a^				
		SUP 1&2	Care 1&2	Care 3–5	Missing	V
Control over daily life		(*n* = 317)	(*n* = 454)	(*n* = 251)	(*n* = 28)	
	Ideal state	25.55	22.03	15.54	21.43	
	No needs	41.01	45.15	51.79	42.86	
	Some needs	26.81	28.63	28.29	35.71	
	High-level needs	6.62	4.19	4.38	0.00	
	Total	100	100	100	100	0.069
Personal cleanliness		(*N* = 335)	(*N* = 463)	(*N* = 257)	(*N* = 32)	
	Ideal state	35.52	28.94	28.79	28.13	
	No needs	59.40	63.07	63.81	65.63	
	Some needs	4.78	7.13	6.23	3.13	
	High-level needs	0.30	0.86	1.17	3.13	
	Total	100	100	100	100	0.055
Food & drink		(*N* = 330)	(*N* = 460)	(*N* = 260)	(*N* = 30)	
	Ideal state	36.97	26.52	23.46	40.00	
	No needs	53.33	60.65	66.92	40.00	
	Some needs	8.48	11.09	7.31	16.67	
	High-level needs	1.21	1.74	2.31	3.33	
	Total	100	100	100	100	0.087
Personal safety		(*N* = 336)	(*N* = 461)	(*N* = 260)	(*N* = 31)	
	Ideal state	26.79	32.54	38.08	32.26	
	No needs	50.30	46.20	48.46	45.16	
	Some needs	19.64	18.22	11.54	22.58	
	High-level needs	3.27	3.04	1.92	0	
	Total	100	100	100	100	0.069
Social participation		(*N* = 334)	(*N* = 462)	(*N* = 250)	(*N* = 28)	
	Ideal state	12.57	9.52	6.80	28.57	
	No needs	56.29	46.10	44.00	39.29	
	Some needs	23.35	35.93	36.00	25.00	
	High-level needs	7.78	8.44	13.20	7.14	
	Total	100	100	100	100	0.108
Occupation		(*N* = 332)	(*N* = 455)	(*N* = 242)	(*N* = 25)	
	Ideal state	21.08	12.75	11.16	28.00	
	No needs	45.78	40.00	29.34	36.00	
	Some needs	27.11	36.92	34.30	36.00	
	High-level needs	6.02	10.33	25.21	0	
	Total	100	100	100	100	0.158
Question item	Scale level	Care level group^a^				
		SUP 1&2	Care 1&2	Care 3–5	Missing	V
Accommodation cleanliness		(*N* = 332)	(*N* = 459)	(*N* = 258)	(*N* = 29)	
	Ideal state	23.19	19.83	28.68	20.69	
	No needs	61.14	64.05	64.73	62.07	
	Some needs	12.95	14.60	6.20	17.24	
	High-level needs	2.71	1.53	0.39	0	
	Total	100	100	100	100	0.083
Dignity filter question		(*N* = 318)	(*N* = 442)	(*N* = 244)	(*N* = 26)	
	Ideal state	23.90	20.59	17.21	30.77	
	No needs	59.43	60.18	55.33	61.54	
	Some needs	14.47	18.55	25.41	7.69	
	High-level needs	2.20	0.68	2.05	0	
	Total	100	100	100	100	0.079
ASCOT Dignity question		(*N* = 322)	(*N* = 440)	(*N* = 281)	(*N* = 26)	
	Ideal state	31.99	22.73	22.41	34.62	
	No needs	54.97	59.09	53.11	53.85	
	Some needs	12.11	17.05	22.82	11.54	
	High-level needs	0.93	1.14	1.66	0	
	Total	100	100	100	100	0.081

*SUP* Support level, *V* Cramer’s V, ^a^Care level groups under Japanese social care system

Table 6 shows the polychoric correlation for all item pairs. The correlations are mostly moderate, ranging between 0.3 and 0.5. *Dignity* shows weak (below 0.3) correlations with *personal cleanliness and comfort, food and drink*, *personal safety*, and *accommodation cleanliness and comfort*. The EFA found the contribution of the first factor to be 41.5%. This exceeded the expected criteria, thus, to examine the scale system we performed a one-factor IRT analysis. Cronbach’s alpha for the whole set of items was 0.794 (95% Confidence interval: 0.773, 0.813), indicating a ‘good’ level.

**Table 6:** Polychoric correlations for pairs of ASCOT item

Question item	Matrix of polychoric correlations							
	Control over daily life	Personal cleanliness	Food & drink	Personal safety	Social participation	Occupation	Accommodation cleanliness	Dignity
Control over daily life	1.000							
Personal cleanliness	0.476	1.000						
Food & drink	0.464	0.464	1.000					
Personal safety	0.391	0.374	0.312	1.000				
Social participation	0.391	0.428	0.381	0.424	1.000			
Occupation	0.443	0.458	0.397	0.394	**0.654**	1.000		
Accommodation cleanliness	0.425	**0.548**	0.441	0.455	0.373	0.345	1.000	
Dignity	0.339	0.288	0.228	0.229	0.341	0.362	0.277	1.000

Data in **bold** are above 0.5

**Fig. 1:**
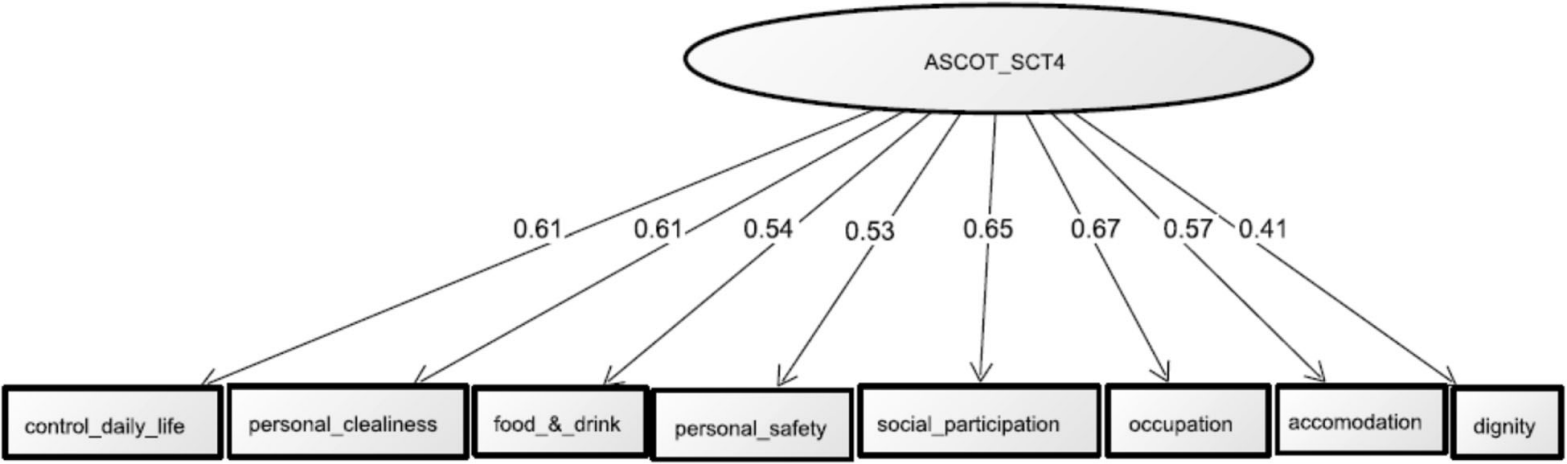
Path diagram

Table 7 shows factor loadings and standard error. The CFA factor loadings ranged from 0.706 (*occupation*) to 0.550 (*dignity*) (all *p* < 0.001). The standard errors ranged from 0.040 (*dignity)* to 0.025 (*occupation*). Figure 1 shows the path diagram. The CFA found that all standardized path coefficients in the model were statistically significant (all *p* < 0.001), showing 0.607 for *control over daily life*, 0.613 for *personal cleanliness*, 0.538 for *food and drink*, 0.531 for *personal safety*, 0.648 for *social participation*, 0.667 for *occupation*, 0.570 for *accommodation cleanliness*, and 0.405 for *dignity*. The values for the model fit were as follows: 0.923 for CFI, 0.910 for TLI, and 0.083 (95% CI: 0.069, 0.098) for RMSE.

**Table 7:** Factor loadings and standard error

Question item	Factor loadings	Standardized error
Control over daily life	0.654	0.027
Personal cleanliness	0.675	0.030
Food & drink	0.579	0.031
Personal safety	0.561	0.030
Social participation	0.705	0.026
Occupation	0.706	0.025
Accommodation cleanliness	0.620	0.031
Dignity	0.550	0.040

**Fig. 2:**
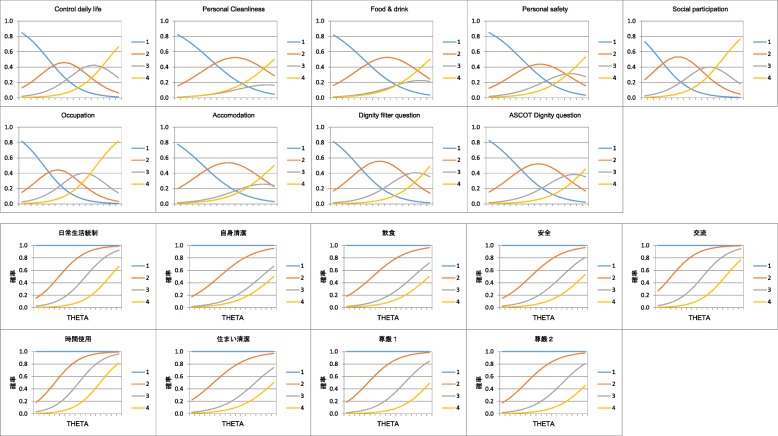
Individual IRCCC for the scale level

Table 8 shows the item discrimination and item difficulty values for the scale levels for each item. All values were within the expected ranges. An exception was observed in the item difficulty for the ‘high-level needs’ level for the ASCOT *dignity* item, showing slightly higher value (4.252) than the expected value (4.0). Figure 2 presents the individual IRCCC for the scale levels within each item. The IRCCC for the *social participation and involvement* and *occupation* items displayed curves shifted neither to the right nor the left. IRCCC for the other items displayed curves slightly shifted to the right. For six items -- *personal cleanliness and comfort*, *food and drink*, *personal safety*, *accommodation cleanliness and comfort*, and two *dignity* question items -- the ‘some needs’ level did not discriminate well.

**Table 8:** Item discrimination and item difficulty for the scale levels

Question item	Item discrimination		Item difficulty					
			‘no needs’ scale		‘some needs’ scale		‘high level needs’ scale	
	EST	SE	EST	SE	EST	SE	EST	SE
Control over daily life	0.922	0.067	−1.151	0.112	0.665	0.097	0.243	0.209
Personal cleanliness	0.975	0.080	−0.704	0.104	2.142	0.184	3.639	0.364
Food & drink	0.757	0.062	−0.902	0.094	2.003	0.120	3.733	0.255
Personal safety	0.723	0.057	−0.769	0.084	1.446	0.102	3.435	0.213
Social participation	1.060	0.078	−1.458	0.168	0.340	0.097	1.851	0.182
Occupation	1.063	0.076	−1.407	0.145	0.192	0.095	1.641	0.163
Accommodation cleanliness	0.841	0.069	−1.140	0.109	1.696	0.130	3.541	0.282
Dignity filter question	0.774	0.076	−1.310	0.114	1.353	0.114	3.799	0.283
ASCOT Dignity question	0.701	0.073	−1.132	0.102	1.561	0.109	4.252	0.314

*EST* Estimation, *SE* Standard error

### Creating the final Japanese version

Table 9 shows the descriptions and translation examples from the final Japanese version of ASCOT SCT4. The main refinements were performed for the *control over daily life* and *dignity* items. For the *control over daily life* item, ‘How much are you able to decide by yourself in terms of your daily life?’ was employed. For the *dignity* items, ‘How do you think and feel about yourself in terms of getting care and support’ was employed. The full approved questionnaire is available on the ASCOT website (www.pssru.ac.uk/ascot). The independent committee commented that some sentences in the questions were repeated in the response options and suggested a revision. However, the Japanese research team, guided by the concept elaboration guide, decided not to revise this, thus the repetitions in the two *dignity* domain questions were retained.

**Table 9:** Descriptions and translation examples [©PSSRU at the University of Kent]

Question item	Description and translation of the items *control over daily life* and two *dignity* question items
Control over daily life	The service user can choose what to do and when to do it, having control over his/her daily life and activities. **Original version** Which of the following statements best describes how much control you have over your daily life? By ‘control over daily life’ we mean having the choice to do things or have things done for you as you like and when you want. I have as much control over my daily life as I want. I have adequate control over my daily life. I have some control over my daily life but not enough. I have no control over my daily life. **The final Japanese version** あなたは, 日常生活において, 自分のことをどのくらい自分で決められていますか.決めたことを, 他の人にやってもらう場合も含めて, お答えください. 思い通り好きなように自分で決められている. おおむね自分で決められている. あまり自分で決められない. まったく自分で決められない.
Personal cleanliness and comfort	The service user feels he/she is personally clean and comfortable and looks presentable or, at best, is dressed and groomed in a way that reflects his/her personal preferences.
Food and drink	The service user feels he/she has a nutritious, varied and culturally appropriate diet with enough food and drink he/she enjoys at regular and timely intervals.
Personal safety	The service user feels safe and secure. This means being free from fear of abuse, falling or other physical harm.
Social participation and involvement	The service user is content with their social situation, where social situation is taken to mean the sustenance of meaningful relationships with friends, family and feeling involved or part of a community should this be important to the service user.
Occupation	The service user is sufficiently occupied in a range of meaningful activities whether it be formal employment, unpaid work, caring for others or leisure activities.
Accommodation cleanliness and comfort	The service user feels their home environment, including all the rooms, is clean and comfortable.
Dignity	The negative and positive psychological impact of support and care on the service users’ personal sense of significance.
	**Dignity filter question** **Original version** Which of these statements best describes how having help to do things makes you think and feel about yourself? Having help makes me think and feel better about myself. Having help does not affect the way I think or feel about myself. Having help sometimes undermines the way I think and feel about myself. Having help completely undermines the way I think and feel about myself. **The final Japanese version** ケアや支援を受けることを, あなたはどのように感じていますか. ケアや支援を受けることで, 今の自分をより良く思える. ケアや支援を受けることは, 自分が自分をどう感じるかとは関係がない. ケアや支援を受けることで, 気持ちが傷つくことがある. ケアや支援を受けることで, 気持ちがひどく傷ついている.
	**ASCOT Dignity question** **Original version** Which of these statements best describes how the way you are helped and treated makes you think and feel about yourself? The way I’m helped and treated makes me think and feel better about myself. The way I’m helped and treated does not affect the way I think or feel about myself. The way I’m helped and treated sometimes undermines the way I think and feel about myself. The way I’m helped and treated completely undermines the way I think and feel about myself. **The pre-final Japanese version** ケアや支援のされ方について, あなたはどのように感じていますか. ケアや支援のされ方により, 今の自分をより良く思える. ケアや支援のされ方は, 自分が自分をどう感じるかとは関係がない. ケアや支援のされ方により, 気持ちが傷つくことがある. ケアや支援のされ方により, 気持ちがひどく傷ついている.

## Discussion

This study identified some cultural challenges during the translation process to develop a Japanese version of the ASCOT-SCT4. The cultural challenges were emerged for the *control over daily life* and dignity items. The direct translation of ‘control’ was not fully accepted by the participants in the cognitive debriefing and the independent committee. The concept of ‘control’ is supposed to capture elements of decision-making, autonomy, and choice where individuals receive care [2, 8, 9]. While the concept of ‘control’ capture these qualities of social care for the study researchers, it was, however, difficult to translate it directly using only one word in Japanese, especially for older adults. Since Asian cultures are collectivist, decision-making involves taking into account opinions of family members. Western cultures, meanwhile, focus more on the individual’s personality and independence, therefore, decision-making is the individual’s responsibility [32]. Furthermore, collectivism and harmony are valued more than asserting one’s opinions over the process of decision-making in Asian cultures [33]. We suggest that it is for this reason that participants found the expression ‘control over’ and the word ‘control’ difficult to paraphrase in the cognitive debriefing.

In recent years, the capacity to have control over one’s life has become more valued in Japan. This has its roots in the Western culture that values an individual’s capacity to exert control over relevant factors which influence decision-making in order to improve QoL [34]. Nevertheless the phrase ‘control over daily life’ seems so far to have been used only in academic reports, not questionnaires and interviews. Indeed, the Japanese version of the health locus of control scale employs the word ‘control’ for the scale name but none of the questions use the word ‘control’ [35]. A different study asked community-dwelling Japanese who were older than 97 years old ‘Have you lived your life as your wished?’ in order to explore ‘perceived control of life’ [36], implying that ‘control’ was not valued strongly but highly. This question ‘How much are you able to decide by yourself in terms of your daily life?’ in the final Japanese version seemed to be acceptable to Japanese older adults.

The word ‘dignity’ is used in Japan to both refer the decision-making and satisfaction. These matters are enshrined in the Japanese constitution [37]. The word is empathized in the context of end of life care [37, 38]. Consequently, we surmise the participants in the cognitive debriefing may have understood the word ‘dignity’ regarding decision-making and satisfaction. Participants in the cognitive debriefing said that they did not use the word for describing daily activities and they use it for describing end of life care.

Alternatively, we employed ‘self-esteem.’ However, cognitive debriefing revealed that the word ‘self-esteem’ was difficult to understand and/or paraphrase for four out of five participants. Moreover, the independent committee fed back that the word had various meanings and could be misunderstood. As a result, the final Japanese version employed the same expression as the English version, i.e. ‘what do you think and feel. ’ (The English version includes neither the word ‘dignity’ nor ‘self-esteem’ in the question sentences and response options for the two dignity question items.)

The translation and cross-cultural validation process has shown the importance of choosing expressions that convey respect for older adults. Like some other Eastern Asian countries Confucian philosophy is important in Japan and influences social norms and values. Confucian philosophy teaches the virtue of respect for elders [39, 40]. The cognitive debriefing and the feedback from the independent committee were helpful for revising the translation so it sounded natural to social care service users and, essentially, for adjusting words and expressions to fit with the language used by the older generations.

The main contribution of this study was the development of a Japanese version of the ASCOT-SCT4 to measure Social-Care Related Quality of Life. In the cross-cultural validation, the factorial structure of the Japanese version of the ASCOT-SCT4 was confirmed as all model fit indices were at acceptable levels. The inter-item polychoric correlations for each domain were all mostly moderate. The internal consistency was acceptable. The results from the factor analysis and the values for polychoric correlation in this study were similar to those with the English data [7]. This study identified that the ‘some needs’ scale did not work well in some question items. For some participants in this study, the degree of ‘some’ may be abstract and difficult to decide what amount is ‘some.’ Providing examples can help participants think in a more concrete way. As discussed above, the items *control over daily life* and *dignity* were difficult to translate and the initial translations were not acceptable to participants in the cognitive debriefing, thus it was important but very time-consuming to revise these items. It was notable that the Dutch translation of the ASCOT-SCT also found these two domains difficult to translate, suggesting those concepts may be very differently expressed across culture.

Evidence of cross-cultural validation is extremely important for ensuring the use of the ASCOT-SCT4 in Japan. At present, Japanese social care levels are assessed by health care professionals, according to applicants’ functions and focus solely on performing ADLs and IADLs [1]. The perceptions of social care service users with respect to areas of their life are not gathered. Yet, all ASCOT domains are considered to be important for adults receiving social care, and are measured by professionals’ opinions in Japan. For instance, food and drink is assessed in terms of nutritional intake by dietitians and public health nurses; however, an individual’s satisfaction in relation to food and drink is not currently measured by standard instruments. The ASCOT-SCT4, would complement the data currently collected and will provide a more comprehensive view of the quality of life of service users from the perspective of the service user. This information will be useful for community-based health professionals and other stakeholders to strengthen the responsiveness, effectiveness and potential cost-effectiveness of services.

### Strength and limitation

This study is the first study to report on the development of a Japanese version of the ASCOT-SCT4 using the approved translation process with cross-cultural validation of the resultant measure among a large sample of older community-dwelling users of social care services. There were some limitations to this research. Only the participants for the cognitive debriefing were recruited based on a theoretical sampling method. The participants for the cross-cultural validation were not randomly selected. Importantly, the survey was on the pre-final Japanese version and it would be of value to conduct similar analysis on the final version.

## Conclusions

This study developed for the first time a Japanese version of the ASCOT-SCT4 instrument for measuring social care related quality of life of social care service users. Our goal in translating ASCOT into Japanese was to support the provision of high quality social care services in Japan by facilitating their appropriate evaluation. The development of the Japanese version of ASCOT-SCT4 is a first step towards meeting this goal. In future research we plan to establish a system for weighting the measure so that the weights reflect the value of the attribute-levels for the Japanese population with anchoring to the dead state. Once the weighting system is established the Japanese version of ASCOT-SCT4 can be used for research into the quality and management of social care services in Japan. The final approved version is accessible at the ASCOT website: https://www.pssru.ac.uk/ascot. Japanese readers may also refer to a report from the translation [41].

## Abbreviations

ADLs: Activities of daily living
ASCOT: Adult Social Care Outcomes Toolkit
IADLs: Instrumental Activities of Daily Living
QoL: Quality of Life
SCRQoL: Social-Care Related QoL
SCT4: Four-level Self-Completion questionnaire
